# Research Recommendations Following the Discovery of Pain Sensitizing IgG Autoantibodies in Fibromyalgia Syndrome

**DOI:** 10.1093/pm/pnab338

**Published:** 2021-11-26

**Authors:** Andreas Goebel, David Andersson, Chris Barker, Neil Basu, Craig Bullock, Stuart Bevan, Rachael J M Bashford-Rogers, Ernest Choy, David Clauw, Debra Dulake, Richard Dulake, Herta Flor, Marcia Glanvill, Zsuzsanna Helyes, Sarosh Irani, Eva Kosek, Jennifer Laird, Gary MacFarlane, Hayley McCullough, Andrew Marshall, Robert Moots, Serge Perrot, Nick Shenker, Emanuele Sher, Claudia Sommer, Camilla I Svensson, Amanda Williams, Geoff Wood, Emma R Dorris

**Affiliations:** Institute of Life Course and Medicine Sciences, Pain Research Institute, University of Liverpool, and Walton Centre NHS Foundation Trust, Liverpool, UK; Institute of Psychiatry, Psychology and Neuroscience, Wolfson Centre for Age Related Disease, King’s College, London, UK; Lancashire and South Cumbria NHS Foundation Trust, UK; Institute of Infection, Immunity & Inflammation, University of Glasgow, Glasgow, UK; Versus Arthritis, Copeman House, St Mary’s Court, St Mary’s Gate, Chesterfield, UK; Institute of Psychiatry, Psychology and Neuroscience, Wolfson Centre for Age Related Disease, King’s College, London, UK; Wellcome Centre for Human Genetics, Oxford, UK; CREATE Centre, Division of Infection and Immunity, Cardiff University, UK; Anesthesiology, Medicine (Rheumatology) and Psychiatry University of Michigan, Ann Arbor, Michigan, USA; Patient Representative, Relative, Mansfield, UK; Patient Representative, Relative, Mansfield, UK; Institute of Cognitive and Clinical Neuroscience, Central Institute of Mental Health, Medical Faculty Mannheim, Heidelberg University, Mannheim, Germany; Patient Representative, Liverpool, UK; Department of Pharmacology and Pharmacotherapy, Medical School & Szentágothai Research Centre, University of Pécs, Pécs, Hungary; Oxford Autoimmune Neurology Group, John Radcliffe Hospital, University of Oxford, Oxford, UK; Department of Clinical Neuroscience, Karolinska Institutet, Stockholm, Sweden; Eli Lilly and Company, Pain & Neurodegeneration Therapeutic Area, Lilly Research Centre, Windlesham, Surrey, UK; Epidemiology Group, Aberdeen, Scotland; Institute of Life Course and Medicine Sciences, Pain Research Institute, University of Liverpool, Liverpool, UK; Institute of Life Course and Medicine Sciences, Pain Research Institute, University of Liverpool, and Walton Centre NHS Foundation Trust, Liverpool, UK; Faculty of Health Social Care and Medicine, Edge Hill University, Liverpool, UK; Liverpool University Hospitals NHS Foundation Trust, Liverpool, UK; Pain Center, Cochin Hospital, Paris University, Paris, France; Rheumatology Research Unit, Addenbrooke’s Hospital, Cambridge, UK; Eli Lilly and Company, Pain & Neurodegeneration Therapeutic Area, Lilly Research Centre, Windlesham, Surrey, UK; Department of Neurology, University Hospital Würzburg, Germany; Department of Physiology and Pharmacology, Center for Molecular Medicine, Karolinska Institutet, Stockholm, Sweden; Health Psychology, UCL Research Department of Clinical, Educational & Health Psychology, University College London, UK; Cambridge Institute for Medical Research, Cambridge, UK; School of Medicine, University College Dublin, Ireland

**Keywords:** Fibromyalgia, Autoantibodies, Research Priorities

## Abstract

**Background:**

Fibromyalgia syndrome (FMS) is the most common chronic widespread pain condition in rheumatology. Until recently, no clear pathophysiological mechanism for fibromyalgia had been established, resulting in management challenges. Recent research has indicated that serum immunoglobulin Gs (IgGs) may play a role in FMS. We undertook a research prioritisation exercise to identify the most pertinent research approaches that may lead to clinically implementable outputs.

**Methods:**

Research priority setting was conducted in five phases: situation analysis; design; expert group consultation; interim recommendations; consultation and revision. A dialogue model was used, and an international multi-stakeholder expert group was invited. Clinical, patient, industry, funder, and scientific expertise was represented throughout. Recommendation-consensus was determined via a voluntary closed eSurvey. Reporting guideline for priority setting of health research were employed to support implementation and maximise impact.

**Results:**

Arising from the expert group consultation (n = 29 participants), 39 interim recommendations were defined. A response rate of 81.5% was achieved in the consensus survey. Six recommendations were identified as high priority- and 15 as medium level priority. The recommendations range from aspects of fibromyalgia features that should be considered in future autoantibody research, to specific immunological investigations, suggestions for trial design in FMS, and therapeutic interventions that should be assessed in trials.

**Conclusions:**

By applying the principles of strategic priority setting we directed research towards that which is implementable, thereby expediating the benefit to the FMS patient population. These recommendations are intended for patients, international professionals and grant-giving bodies concerned with research into causes and management of patients with fibromyalgia syndrome.

## Background

Fibromyalgia syndrome (FMS) is a widespread chronic pain condition associated with multimodal sensory hyperresponsiveness, fatigue, and changes in cognition, which typically affects mood and/or function [1, 2]. The World Health Organization (WHO) ICD-11 has classed FMS as “chronic primary pain.” FMS is common, with an estimated prevalence of between 2% and 6% in the general population worldwide, depending on criteria used [3]. It imposes a major burden on affected individuals, the healthcare system, and the general economy (Silverman et al. 2009). The pathophysiology of FMS is poorly understood, and available treatments are insufficiently effective [4]. An absence of specific disease biomarkers hinders patient stratification and impacts on all areas of research into FMS.

Recently, a consortium of UK and Swedish investigators has reported research findings indicating that noninflammatory immunoglobulin G (IgG) serum autoantibodies (Aab), without a systemically measurable inflammatory response contribute to the pathogenesis of FMS [5]. The team transferred both single and pooled serum-IgG from patients with FMS to rodents and identified typical features of clinical FMS in the rodents, including sensitivity to pressure and cold, reduced grip strength and movement, and small fiber pathology when compared to the transfer of serum-IgG from healthy volunteers. The antibodies were shown to specifically bind to structures within mouse and human dorsal root ganglia. These findings may provide a first pathogenic clue to the pathophysiology of FMS and necessitate questions about how the pain research community can use this new knowledge to leverage and improve research in this area.

In order to address the implications of these findings for FMS research an expert multidisciplinary working group was convened. The aim of this group was to develop patient-centred, translatable, and clinically useful research priority recommendations based on these novel findings in FMS. Here we outline these recommendations and detail their development.

The initiative was developed with the aim of formulating research recommendations arising from the recent discovery of pronociceptive Aab in FMS [5]. We adapted the REPorting guideline for PRIority Setting of health research (REPRISE) [6] to facilitate comprehensive reporting of the development of, and conclusions arising from, the expert working group in FMS.

These recommendations are primarily intended for professionals and grant-awarding bodies internationally concerned with research into the causes and management of patients with FMS, to ultimately benefit this patient population. A revision is planned in 5 years’ time.

## Methods

The reporting of this study follows the REPRISE and CHERRIES guidelines [6, 7].

### Governance and Team

The initiative was developed by one of the authors (AG) after consultation with co-authors in the antibody publication. Additionally, stakeholders were invited that were independent FMS experts or experts in autoantibody research as identified by these authors, as well as the Funder (Versus Arthritis [VA]), the UK’s largest charity dedicated to supporting people with arthritis, rheumatic diseases, and musculoskeletal disorders such as FMS, which also has extensive experience in research priority initiatives https://www.versusarthritis.org/. Senior functional leaders from Eli Lilly, a large pharmaceutical company with an established autoimmune and pain portfolio were invited to contribute industry stakeholder perspectives. Administrative support was provided through a VA administrator, and an administrator funded via the Pain Relief Foundation, Liverpool, a dedicated UK Pain Research charity https://painrelieffoundation.org.uk/ (HMC). A researcher independent from VA with experience in leading research priority setting exercises (ED) supported the output write-up.

### Public and Patient Involvement

Versus Arthritis was involved throughout this project. In addition, two people living with FMS and one familial carer were members of the working group. These patient research partners were invited from participants in an existing FMS patient involvement initiative at the University of Liverpool, and from amongst a patient involvement group within VA. They took part in all discussions, feedback, consensus survey and reviewed the manuscript. Patient research partners were offered assistance with the consensus survey, including assistance with the language, terminology, and technology used.

### Process Framework

We followed a dialogue model [8] with main principles including respect for the experiential knowledge of all the participating stakeholders. The focus of the dialogue model is improved stakeholder learning opportunities through dialogic processes that explore and move beyond initial assumptions about facts and values surrounding complex problems. Briefly, through the process of i) situation analysis, ii) design, iii) expert group consultation, iv) interim recommendations, and v) consultation and revision (outlined in greater detail in the next section) stakeholders learn from each other the different ways that a shared problem can be defined through reframing and reformulating stakeholder issues, problems, and questions. This emphasizes mutual learning between stakeholders of all types and enhances shared understanding of the issue at hand for all stakeholders.

#### Research Recommendation Process

**Figure 1.**
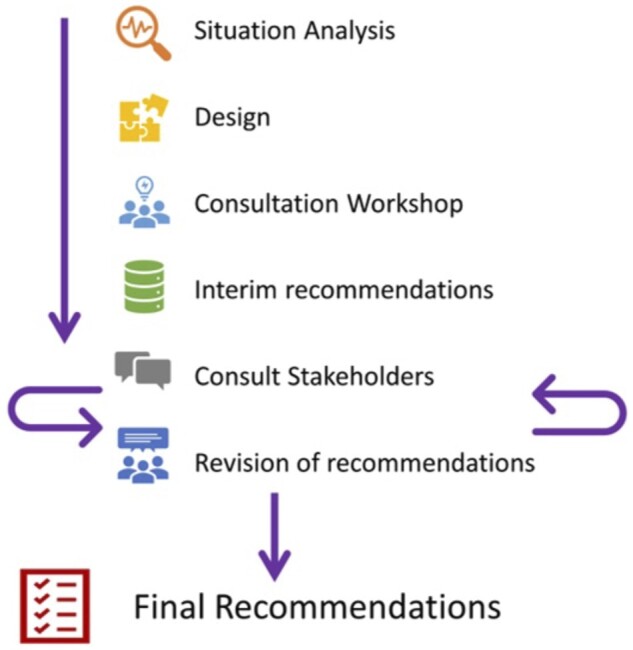
Research recommendation process workflow.

The initiative was conducted in five phases, i) situation analysis, ii) design, iii) expert group consultation, iv) interim recommendations, and v) consultation and revision (Figure  1).

Situation analysis was conducted by the convenors defining the scope. Design phase included stakeholder engagement, protocol development, and defining decision making criteria. The expert group consultation is outlined in detail below. This consisted of a 2-day face-to-face workshop of invited multidisciplinary researchers, clinicians and people living with FMS. It was facilitated by the research charity funders (VA). Interim recommendations arising from the workshop were prepared and shared with the stakeholders who had attended the meeting. Stakeholders were invited to critically assess these recommendations, which were reviewed in an iterative manner until consensus was reached. After two iterative rounds, an anonymous e-survey was conducted to ascertain levels of stakeholder agreement.

#### Stakeholders/Participants

The group consisted of 29 stakeholders (16 male, 13 female), of which n = 25 also took part in the face-to-face workshop; it included patients (n = 2) and caregivers (n = 1). One of the participating patients had previously received specific research review training from VA and had experience as a “patient insight partner” for pain-related research. The group provided a broad mix of clinical and scientific skills and experiences (Table  1). Additionally, stakeholders’ countries of residence had a wide geographic spread (UK 21 of which England 18, Scotland 2, Wales 1, France 1, Germany 2, Hungary 1, Ireland 1, Sweden 2, USA 1). These were 27 established researchers or consultant level clinicians and two early career researchers. These participating researchers were included based on their relevant experience, including expertise in Aab research, Aab contribution to FMS, the mechanisms underpinning rheumatological or neurological autoimmune disorders, or a track record of research activity in fibromyalgia and/or chronic pain. Three representatives from the research department of a pharmaceutical company with an active pain portfolio also took part; two staff members of the Funder’s research and discovery department participated in the workshop, and additionally a VA administrator (as a nonstakeholder). All stakeholders were invited to contribute to defining the final meeting agenda. All travel expenses were paid to the nonindustry participants, as was overnight accommodation if needed, no honoraria were offered.

**Table 1.** Stakeholder professional background

	Workshop Stakeholders		Active Healthcare Professional	
	n	% Total	N	% Total
Person living with FMS	2	7	…	
Carer for person living with FMS	1	3.5	…	
Rheumatologist	7	24	7	50
Pain specialist	1	3.5	1	7
Psychologist	2	7	1	7
Geneticist	1	3.5	1	7
Epidemiologist	1	3.5	…	
General practitioner	1	3.5	1	7
Clinical neurophysiologist	1	3.5	1	7
Academic pharmacologist	1	3.5	…	
Neurologist	2	7	2	14
Cellular immunologist	1	3.5	…	
Molecular biologist	2	7	…	
Citizen engagement researcher	1	3.5	…	
Pharmaceutical representatives	3	10	…	
Charity funder R&D	2	7	…	
Total	29		14	

FMS = fibromyalgia syndrome.

#### Situation Analysis and Consultation Design

A draft content outline of potential research topics that may need discussion was formulated by AG integrating similar topics addressed in other autoimmune conditions and modelled on previous focus groups on immune trials in chronic pain conditions and multi-stakeholder processes in pain conditions [9, 10]. This outline and a preliminary list of stakeholders was circulated among two of these stakeholders (D.A., C.B.) who made suggestions about content and involvement of additional stakeholders; a revised content proposal was sent to the identified stakeholders and changes (both regarding content and invitation of additional stakeholders) were implemented and a final version circulated amongst all stakeholders. An agenda for a two-day workshop was condensed from that document; the agenda was circulated in advance to allow a wider stakeholder review, and revisions were implemented where necessary. The finalized agenda included 11 topic areas for discussion.

The aim for the workshop was to include discussion of the full range of suggested topic areas without any ranking or exclusion. The 11 topic areas are outlined in Table  2. At the face-to-face workshop each topic area was introduced by one stakeholder with expertise in that field, and then deliberated with the working group using a roundtable discussion format.

**Table 2.** Workshop discussion topics

1.	Overview of the current state of what is known about FMS, with relevance for Aab research.
2.	Preclinical research into Aab mechanisms in FMS.
3.	The role of B-cells in FMS.
4.	Genetic underpinnings of FMS.
5.	Is there a relationship between small fibre neuropathy and pronociceptive Aab in FMS?
6.	Assessment of functional brain activation and research into brain-targeting interventions.
7.	Evolutionary underpinnings of autoimmunity in FMS.
8.	Potential new or adapted clinical treatments for FMS in light of Aab findings.
9.	Trial design for FMS trials.
10.	Research recommendations to investigators in independent trials in other conditions, to capture prevalence and response of comorbid FMS.
11.	How patient involvement can enhance research.

Aab = autoantibody; FMS = fibromyalgia syndrome.

#### Analysis and Identification of Research Recommendations

Minutes from the working group meeting were taken independently by three people. These minutes were then combined. In preparing the overall meeting minutes, the extensiveness, intensity, and specificity of comments at the meeting were considered. Draft minutes were circulated to the entire working group for review to minimise bias and ensure they accurately reflected the working group discussion. The minutes were used to identify research recommendations across all 11 topics discussed. Recommendations were identified. These recommendations were grouped into seven broad areas (Table  3). Feedback and consensus on these 39 recommendations were assessed via an online survey.

**Table 3.** Research recommendation categories

Category	Topic	Number of Recommendations
1.	Facets of fibromyalgia	7
2.	Aab research	7
3.	Immunology	10
4.	Small fiber neuropathy	3
5.	Clinical trials	5
6.	Clinical trial design	5
7.	Evolutionary aspects	2

Aab = autoantibody.

#### Consensus eSurvey

Quota sampling of the working group was used for a voluntary closed survey hosted on the survey monkey platform. The survey consisted of 40 questions. The survey was distributed across nine pages. Page 1 was the introduction; pages 2–8 were the 39 recommendations (one category per page), and page 9 was a thank you and a “further comments” question. Participants could review and change their answers. The research recommendations were assessed via a 5-point Likert scale (Strongly Agree; Agree; Neither Agree nor Disagree; Disagree, and Strongly Disagree).

The eSurvey was reviewed by one native and one non-native English speaker. Participants were informed of the intended purpose of the survey and that it was anonymous. The survey was open for 20 days, starting on 23 April 2020. It was advertised via email to the working group members. Three emails were sent, each four days apart, to encourage participation. The survey link was included in each email. No incentives were offered for completion. Unique survey respondents were measured via IP address. Cookies were not used.

#### Survey Analysis

An IP check was performed to identify potential duplicate entries. No duplicate entries were identified. All questionnaires, including incomplete questionnaires, were analyzed. “Strongly agree” and “agree” responses were combined as “agreement,” and all other responses were considered as disagreement. Data were coded and SPSS (v24.0) was used to preform frequency analysis. Agreement data are reported as count (n), percentage agreement of respondents, and percentage agreement of total cohort.

## Results

Two members of the working group self-selected out, to avoid potential perceived bias (one funder and one pharmaceutical industry representative). The cohort was n = 27. Of these, n = 22 responded, giving a response rate of 81.5%. As 81.5% was the highest achievable percentage of agreement for the total cohort, the consensus for each recommendation was categorised based on percentage agreement of total cohort: high consensus (>72%), medium (62%–71%), low (51–61%), and less than 51% was deemed to have no consensus. Six recommendations achieved high consensus, whereas n = 15 had medium level of consensus (Table  4).

**Table 4:** Results of research recommendation consensus

Category	Question	Agree (n)	% Respondents	% Working Group	Level of Consensus
1	Due to heterogenicity of FMS, all research should specify how FMS was defined and how specific phenotyping criteria were used.	22	100.00	81.48	HIGH
2	Replication studies of the Aab findings by independent researchers and research labs are recommended.	21	95.45	77.78	HIGH
2	Characterisation of the Aab target(s) is recommended.	21	95.45	77.78	HIGH
3	Immune response during FMS flares versus nonflare should be investigated.	21	95.45	77.78	HIGH
1	Experimental modelling of poly-sensory hyper-responsiveness and other FMS-related features (such as fatigue and cognitive problems) are relevant to gain better understanding of any Aab contribution.	20	90.91	74.07	HIGH
6	Primary endpoints for trials should reflect the multidimensional experience of fibromyalgia, rather than only the perceived pain intensity.	20	90.91	74.07	HIGH
1	Research needs to address the question of how chronic overlapping pain condition components and/or any overlapping phenotypes may be specifically contributed to by Aab (examples of COPS include chronic pelvic pain, painful bladder syndrome, irritable bowel syndrome, low back pain, burning mouth syndrome, chronic post-traumatic pains, chronic fatigue syndrome, and others).	19	86.36	70.37	MED
1	Classification of FMS sub-phenotypes based on clinical presentation, treatment responses or familiar clusters and association with genetic variants related to immune function should be investigated.	19	86.36	70.37	MED
2	Research should address how Aab can cause central phenomena in FMS.	19	86.36	70.37	MED
2	The Aab binding in the central nervous system should be further investigated.	19	86.36	70.37	MED
4	The hypothesis that Aab induce abnormal nerve fibre activation independent of any structural nerve fibre change should be investigated.	19	86.36	70.37	MED
6	Trial designs should be co-created with patients and interdisciplinary professionals.	19	86.36	70.37	MED
1	Aab research should take into account sub-threshold ‘fibromyalgianess’ and the wide prevalence of this problem, rather than exclusively focus on ‘above threshold’ fibromyalgia.	18	81.82	66.67	MED
3	The overall antibody subclass levels in FMS patient and associations between FMS and immunoglobulin subclasses or subclass ratios should be investigated.	18	81.82	66.67	MED
3	Longitudinal studies investigating Aab serum levels before and after behavioural-, brain-based-, physiotherapy- or drug-mediated interventions should be conducted.	18	81.82	66.67	MED
4	The relationship between small fibre neuropathy in FMS and the presence of pain-sensitising Aab should be investigated.	18	81.82	66.67	MED
6	Research into decision making of people with FMS on participation in clinical trials where pain relief is a possible outcome should be conducted to develop methods towards ensuring that informed decisions can be made especially where trial interventions have potential serious adverse effects.	18	81.82	66.67	MED
2	For each identified Aab target, the direct antibody pathogenicity versus epiphenomenon should be investigated.	17	77.27	62.96	MED
3	The mechanisms underpinning the production of non-inflammatory Aab in FMS should be investigated.	17	77.27	62.96	MED
5	A three-arm experimental trial including IgG immunoadsorption versus plasma exchange should be considered with the objective of understanding the clinical relevance of pain-sensitising Aab in FMS.	17	77.27	62.96	MED
6	Placebo-dread (patient fear of reporting pain relief and subsequently finding out they were randomised to the placebo group) should be specifically addressed in the FMS trial protocols.	17	77.27	62.96	MED
1	Functional brain risk biomarkers (such as after the experience of life events before the development of pain) should be investigated in the context of serum Aab prevalence.	16	72.73	59.26	LOW
2	Understanding of Fab-mediated vs. Fc mediated effector processes is recommended.	16	72.73	59.26	LOW
3	Once target epitopes are known, the transfer of pertinent, pathogenic B-cells should be investigated.	16	72.73	59.26	LOW
3	Upon identification of epitopes, the effect of either prior or current immune therapies on pathogenic B-cell clones should be investigated.	16	72.73	59.26	LOW
3	Epigenetic studies, and association with environmental factors that trigger Aab production, should be investigated.	16	72.73	59.26	LOW
5	Intervention trials in conditions with co-morbid FMS should facilitate FMS and fibromyalgianess assessment at baseline and the primary endpoint to capture vital ‘planned serendipity’ data about the effect of the respective intervention in FMS.	16	72.73	59.26	LOW
6	The natural fluctuation of FMS symptoms, such as with menstruation, should be addressed as part of FMS trial designs.	16	72.73	59.26	LOW
2	The biophysical properties of Aab should be investigated (including pathogenicity, affinity, electrical charge).	15	68.18	55.56	LOW
3	Bulk B-cell or T-cell receptor repertoire analysis in FMS should be investigated.	15	68.18	55.56	LOW
4	Proximal skin-nerve density in the rodent model should be studied given recent clinical results related to small fibre neuropathy in FMS.	14	63.64	51.85	LOW
5	An experimental trial of plasma exchange in FMS should be considered.	14	63.64	51.85	LOW
7	The hypothesis that Aab production in FMS is a maladaptive by-product of pain mechanisms and neural plasticity should be investigated.	14	63.64	51.85	LOW
3	Evidence of maternal-foetal/infant transfer of hypersensitivity in the third trimester, or through breast-feeding should be investigated.	11	50.00	40.74	NO
3	Association of FMS with primary immunodeficiencies should be investigated.	11	50.00	40.74	NO
7	Research to understand if Aab in FMS convey a selective advantage (e.g., lower susceptibility to certain health problems, higher alertness to environmental stimuli) should be conducted.	11	50.00	40.74	NO
1	Association of Aab with depression and anxiety should be investigated in the rodent FMS models.	10	45.45	37.04	NO
5	Treatments that directly reduce pathogenic antibody levels such as plasma exchange, FcRn modifiers, or T-cell targeting drugs such as abatacepts should be the first line choice for clinical trials.	10	45.45	37.04	NO
5	Results from epitope research and antibody-antigen interaction should be awaited before any experimental trials are considered	8	36.36	29.63	NO

Aab = autoantibody; FMS = fibromyalgia syndrome.

Research recommendations with high or medium stakeholder agreement fell into the following five thematic groups:

- Facets of FMS (category 1, n=5)
- Aab Research (category 2, n=5)
- Immunology (category 3, n=4)
- Small Fibre Neuropathy (category 4, n=2)
- Clinical Interventions and Trial Design (categories 4 and 5, n=5)

## Discussion

### Facets of FMS

This category includes recommendations about aspects of fibromyalgia features and associated symptoms that should be considered in all future Aab research.

1. There was high stakeholder agreement on the necessity to clearly outline diagnostic and phenotyping criteria in any research going forward as, for example, patient populations markedly differ between diagnostic categories such as ACR 2010, ACR 2016 [11].
2. Stakeholders considered that research should attempt to classify FMS subtypes. FMS appears clinically heterogeneous. Antibody prevalence and specificity, along with familiar prevalence, genetic variants, clinical presentations, and treatment responses may support the definition of FMS sub-phenotypes, with implications for clinical management.
3. Antibody research should be broadened:
4. Research should investigate Aab contribution to polysensory hyper-responsiveness and other FMS features including fatigue and cognitive problems, including in the recent rodent ‘transfer’ models where IgG taken from FMS patients is injected to rodents causing abnormalities resembling FMS in these animals [5].
5. The contribution of Aab to chronic pain conditions associated with “*fibromyalgianess*,” *below* the ACR 2016 score threshold for the diagnosis of FMS [12], and in chronic overlapping pain conditions (COPCs) should be investigated. *Fibromyalgianess* is an emerging concept; it describes a symptom complex variably comprised of fatigue, sleep disturbance, cognitive symptoms, poly-sensory hyper-responsiveness, and psychological co-morbidities. Risk factors for this phenotype include female sex, regional or widespread pains and exposure to stressors, but it seems independent of both known peripheral triggers or individual psychopathology [13]. COPCs include conditions such as chronic pelvic pain, painful bladder syndrome, irritable bowel syndrome, low back pain, burning mouth syndrome, post-traumatic chronic pain, chronic fatigue, and others [14]. It is more rule than exception that people with FMS have suffered or are suffering from COPCs, and how antibodies contribute to these pain phenotypes (both with and without presence of FMS) would be important to investigate.

### Aab Research

There was a high level of stakeholder agreement that the recent Aab findings should be repeated by independent researchers and research laboratories. It was acknowledged that the current findings already incorporate some degree of replication from two independent laboratories and two distinct patient cohorts.

Stakeholders considered characterisation of Aab targets as a key relevant objective for Aab research going forward, with a high consensus level. There was also a good level of agreement that for each identified Aab target the antibody pathogenicity should be investigated; it was noted that specific Aab associated with a disease can sometimes be without a clear pathogenetic role.

Stakeholders considered it important to research if and how Aab can cause central phenomena that characterize FMS, and to further investigate Aab binding in the central nervous system (CNS). Stakeholders acknowledge that no evidence for CNS binding has been identified but advised that this may not exclude binding at lower intensities or to distinct, small regions.

### Immunology

There was strong agreement that immune parameters should be assessed during FMS flares versus non-flare periods; the dominant antibody subclasses and their titres should be studied [15]. It was thought possible that key parameters such as antibody levels or plasmablast populations may differ during flare times. This is also a method to facilitate generation of antibodies in vitro, from patient B cells. Stakeholders also agreed that the mechanisms underpinning the production of non-inflammatory Aab should be investigated.

In clinical trials, Aab serum levels—alongside the above immunological parameters—should be ascertained both at baseline and post-intervention; this includes both pharmaceutical, and non-pharmaceutical intervention (e.g., behavioural) trials.

### Clinical Trials

#### Trial Design in FMS Trials

Although not specific to Aab research, stakeholders considered the design of clinical trials; one consideration was that side effect profiles for any immunological drug trials addressing Aab mechanisms would differ from currently used drugs, and trial designs would need to address this.

There was high consensus that *primary* endpoints for interventional trials in FMS should reflect patients’ multi-dimensional experiences, which often include symptoms such as cold sensitivity, pressure sensitivity, emotional distress, and fatigue, rather than solely focusing on the perceived pain intensity [16]. Stakeholders discussed how their consensus is best reconciled with traditional recommendations of using pain intensity as the primary outcome for pain-condition trials. A discussion within the worldwide research community was suggested important.

It was agreed that FMS interventional trial designs should generally be co-created both with patients and interdisciplinary professionals.

Stakeholders agreed that research into decision making by people with FMS about their participation in interventional trials would be important, to employ methods that truly empower patients in their decision making, particularly in the context of drugs which can have serious adverse effects [9, 17].

“Placebo-dread” may affect trial recruitment and outcomes in FMS trials, where patients fear that reporting a good improvement of their symptoms may later be interpreted as being “all in their head” if randomized to placebo; this may be particularly important in investigator-led trials where patients may know the principal investigator from clinical interactions. Such placebo-dread might lead to under-reporting of good outcomes. Little research exists in this area, and more is needed. Stakeholders agreed that this issue should be addressed in FMS trial designs. Good patient preparation before trial entry should go some way to minimise this issue; for example, conveyance of an understanding of the universal nature of the placebo effect (not just limited to FMS patients) may be useful. Independently, it was also noted that longer FMS durations may be associated with lower placebo responses [18].

#### Suggestions for Clinical Trials in FMS

This topic was discussed extensively, and good agreement was found for only one recommendation. Stakeholders agreed that a three-arm *experimental* trial including immunoglobulin G (IgG) immunoadsorption versus plasma exchange should be considered to improve understanding of the clinical relevance of pain-sensitizing Aab in FMS. Patients would need to be told before enrolment that even if that trial was successful these interventions may not become available in clinical practice. Plasma exchange (removal of serum and all solutes from the blood) and immunoadsorption (removal of IgG or IgG subclasses through specific columns) are often effective in Aab-mediated autoimmune conditions [19]. However, these interventions are cumbersome given they require venous access and are generally considered less suited for long-term treatment. They are frequently being used to interrupt disease flares or to rapidly bring an immune disorder under control until the effect of disease modifying treatments set in. There was agreement that FMS patients enrolled in interventional trials aiming to address Aab or their downstream mechanisms should be tested for the presence of such antibodies; serum tests are yet to be developed—passive transfer bioassay testing of each screened patient is unlikely feasible or ethically acceptable.

### Small Fiber Neuropathy

Recently, several research groups have established that small fiber pathology often affect patients with FMS, with a correlation to the FMS disease severity in smaller subgroups [20]. At the same time, nociceptor excitability measured using microneurography is often abnormal [21]. Correspondingly, investigations in the recent FMS rodent-transfer model included assessment of small fibre neuropathy following Aab transfer, with results indicating that dermal nerve fibre density is indeed reduced in the mouse hind paw skin from at least as early as 4 days after transfer start, although behavioural abnormalities are present from at least 1 day after administration of human IgG. This raises the question of whether the abnormal rodent behaviour is secondary to the developing structural nerve fiber abnormalities, or alternatively whether it is due to primarily functional changes induced by the Aab. The latter principle has recently been demonstrated in a different, very rare Aab mediated, painful neurological condition [22]. Stakeholders agreed that the hypothesis that Aab can induce abnormal nerve fibre activation *independent* of any structural nerve fibre change should be further investigated, and also that the relationship between small fibre neuropathy and the presence of pain-sensitizing Aab should be addressed.

### Additional Points of Discussion

Some discussion points not included in the survey were thought relevant by the organising team; in part they relate to matters of fact, or to notes made during the workshop. We outline these here, highlighting that the level of agreement was not assessed:

- The degree of fibromyalgianess is directly associated with unfavourable health outcomes, independent of the diagnosis of FMS [23–26].
- Patients suffering from a higher number of COPCs may have higher fibromyalgianess scores, but this has not been assessed systematically.
- Patients with high symptom severity scores (SSS) may experience more stable FMS than others.
- Clinical assessment of comorbid FMS can support rational treatment; for example, in rheumatoid arthritis may avoid successive biological treatments to address patient pain complaints caused by FMS [27, 28].
- Possible immune contribution in FMS has previously been suggested indirectly by studies assessing the efficacy of IVIG treatment in a subgroup of non-comorbid FMS patients [29, 30], in FMS patients co-morbid with polyneuropathy [31], and by demonstration of abnormal IgG skin deposits in FMS [32].
- The transfer model cannot easily model long-term changes, due to the development of serum sickness in the animals arising from prolonged human protein transfer.
- Antibody profiles, specificities and/or functional effects in FMS differ from those earlier described in other chronic pains conditions, for example Complex Regional Pain Syndrome (CRPS) [33] and non-inflammatory joint pains in RA [34], suggesting that specific Aab may cause specific chronic pain phenotypes.
- Clinical methods to better understand the role of small fibre pathology may include longitudinal investigations, for example, in the context of effectiveness trials (repeat skin biopsies, or corneal microscopy at baseline/follow-up). More information about the relation between small fibre changes and *fatigue* would also be important.
- Anecdotal evidence from patients with co-morbid FMS treated for their rheumatological condition with biologics including anti-cytokine agents, or B-cell therapies suggest that FMS condition may *not* readily respond to these therapies; independently, the situation in axial spondylarthritis may be more complex, as in this condition fulfilling FMS criteria may not necessarily mean that FMS is truly present, and the symptom severity score may be a better measure [35]. More systematic understanding of these issues is required to avoid potentially futile trial efforts.
- New, effective immune interventions in FMS patients, unless achieving complete remission, may challenge trial-patients’ established activity-pacing skills. Patients who have less pain on drug may overdo their activities resulting in pain increase, potentially causing bias in clinical trials. Clinical trials should consider including multidisciplinary support for pacing daily activities at the respective new pain thresholds.
- Multidisciplinary preparation prior to a trial may also allow patients to put symptoms experienced during the trial, both beneficial and adverse, into context, resulting in better adherence and a less stressful experience [17].
- Inclusion of PainDetect [36] and “First” [37] to the outcome questionnaires in FMS trials may provide further relevant information.
- Symptoms and diseases can be understood within an evolutionary framework as arising from adaptive defences [38]; this includes depression [39] and the framework could usefully be applied to the auto-antibody production in FMS

### Limitations

One limitation was that we included relatively fewer early career researchers or trainee clinicians when compared to established researchers. Future processes might enhance inclusiveness and multifaceted responses by including additional participants from the former group.

## Conclusion

Recent findings of pain-sensitising Aab in FMS open a new field of research, with questions extending across a wide range of areas. In this focussed research priority setting exercise, stakeholders from a mix of professional backgrounds and world-regions, funders, relatives, and patients with FMS agreed on a set of research recommendations. These proposed research areas might be taken forward by interested researchers and funders worldwide.

Stakeholders agreed that the confirmation of Aab contributing to the cause of FMS would constitute a transformative finding for this field. It would also represent a revolutionary advance that should remove the perceived stigma arising from the current lack of understanding often communicated by patients [40].

Ensuring that research is centred on improving FMS management and is translatable to a clinical context will be critical to improve the quality of life for this historically undermanaged patient cohort [41].

## Ethics Approval and Consent to Participate

Not applicable.

Consent for publication.

Not applicable.

Availability of data and materials.

The data sets generated and/or analyzed during the current study are available in the Figshare repository, [DOI: 10.6084/m9.figshare.12602186 (will become activated following acceptance for publication)]

## Authors' Contributions

Conception: A.G.

Design of the work:

Acquisition of data: A.G., D.A., C.B., N.B., C.Bk., S.B., R.B.R., E.C., D.C., D.D., R.D., H.F., M.G., Z.H., S.I., E.K., J.L., G.M.F., H.M.C., A.M., R.M., S.P., N.S., E.S., C.S., C.Sn., A.W., G.W., E.D.

Analysis, and interpretation of Data: A.G., E.D.

Drafted the work: A.G., E.D.

All authors have reviewed and approved the manuscript.

## Notes

Conflicts of interest: E.C. has received research grants, honoraria and served as member of advisory boards and speaker bureaus of Abbvie, BioCancer, Biocon, Biogen, Bristol Myer Squibbs, Chugai Pharma, Eli Lilly, Gilead, Janssen, Merck Serono, Novartis, Pfizer, Regeneron, Roche, Sanofi, and UCB. J.L. and E.S. are employees and shareholder of Eli Lilly and Company; SRI is supported by the Wellcome Trust (104079/Z/14/Z), the UCB-Oxford University Alliance, BMA Research Grants- Vera Down grant (2013) and Margaret Temple (2017), Epilepsy Research UK (P1201), the Fulbright UK-US commission (MS-Society research award) and by the NIHR Oxford Biomedical Research Centre. The views expressed are those of the author(s) and not necessarily those of the NHS, the NIHR, or the Department of Health). S.R.I. is a coapplicant and receives royalties on patent application WO/210/046716 (U.K. patent no., PCT/GB2009/051441) entitled ‘Neurological Autoimmune Disorders’. The patent has been licensed to Euroimmun AG for the development of assays for LGI1 and other VGKC-complex antibodies. All other authors declare that they have no potential conflicts.

Funding sources: The project was funded through an educational grant and received administrative support from Versus Arthritis, a UK research charity focussed on improving lives of people living with musculoskeletal conditions https://www.versusarthritis.org/ (total £10,000). Administrative support was provided by the Pain Relief Foundation, Liverpool, a research charity dedicated to supporting research into the causes and treatment of chronic pain https://painrelieffoundation.org.uk/. Z.H. was supported by Hungarian Brain Research Program (20017-1.2.1-NKP-2017-00002, NAP2.0) and EFOP-3.6.2-16-2017-00008.
